# Parthenin—A Sesquiterpene Lactone with Multifaceted Biological Activities: Insights and Prospects

**DOI:** 10.3390/molecules26175347

**Published:** 2021-09-02

**Authors:** Amarpreet Kaur, Shalinder Kaur, Rupali Jandrotia, Harminder Pal Singh, Daizy Rani Batish, Ravinder Kumar Kohli, Virendra Singh Rana, Najam A. Shakil

**Affiliations:** 1 Department of Botany, Panjab University, Chandigarh 160 014, India; amanhayer411@gmail.com (A.K.); rupalijandrotia@gmail.com (R.J.); rkkohli45@yahoo.com (R.K.K.); 2 Department of Environment Studies, Panjab University, Chandigarh 160 014, India; 3 Division of Agricultural Chemicals, Indian Agricultural Research Institute, PUSA, New Delhi 110 012, India; virendra_agchem@iari.res.in (V.S.R.); iamshakil@gmail.com (N.A.S.)

**Keywords:** pharmacological properties, pseudoguaianolide, sesquiterpene lactone, toxicology, terpenoids

## Abstract

Parthenin, a sesquiterpene lactone of pseudoguaianolide type, is the representative secondary metabolite of the tropical weed *Parthenium hysterophorus* (Asteraceae). It accounts for a multitude of biological activities, including toxicity, allergenicity, allelopathy, and pharmacological aspects of the plant. Thus far, parthenin and its derivatives have been tested for chemotherapeutic abilities, medicinal properties, and herbicidal/pesticidal activities. However, due to the lack of toxicity–bioactivity relationship studies, the versatile properties of parthenin are relatively less utilised. The possibility of exploiting parthenin in different scientific fields (e.g., chemistry, medicine, and agriculture) makes it a subject of analytical discussion. The present review highlights the multifaceted uses of parthenin, on-going research, constraints in the practical applicability, and the possible workarounds for its successful utilisation. The main aim of this comprehensive discussion is to bring parthenin to the attention of researchers, pharmacologists, natural product chemists, and chemical biologists and to open the door for its multidimensional applications.

## 1. Sesquiterpene Lactones: An Introduction

Sesquiterpene lactones (STLs) are a diverse group of secondary metabolites of plant origin that are characterised by an array of pharmacological and therapeutic properties and biological activities, including plant-defence abilities, allergenicity, cytotoxicity, and allelopathy [1]. Recent studies indicate that STLs act as signalling compounds in below-ground rhizospheric interactions [2]. Thus far, 5000 STLs have been reported in various angiosperms and bryophytes, but their paramount dominance is in the family Asteraceae, where they are nearly ubiquitous [1,3]. A few well-known and widely studied STLs include artemisinin, parthenolide, helenalin, costunolide, thapsigargin, santonin, and mexicanin [1].

**Figure 1:**
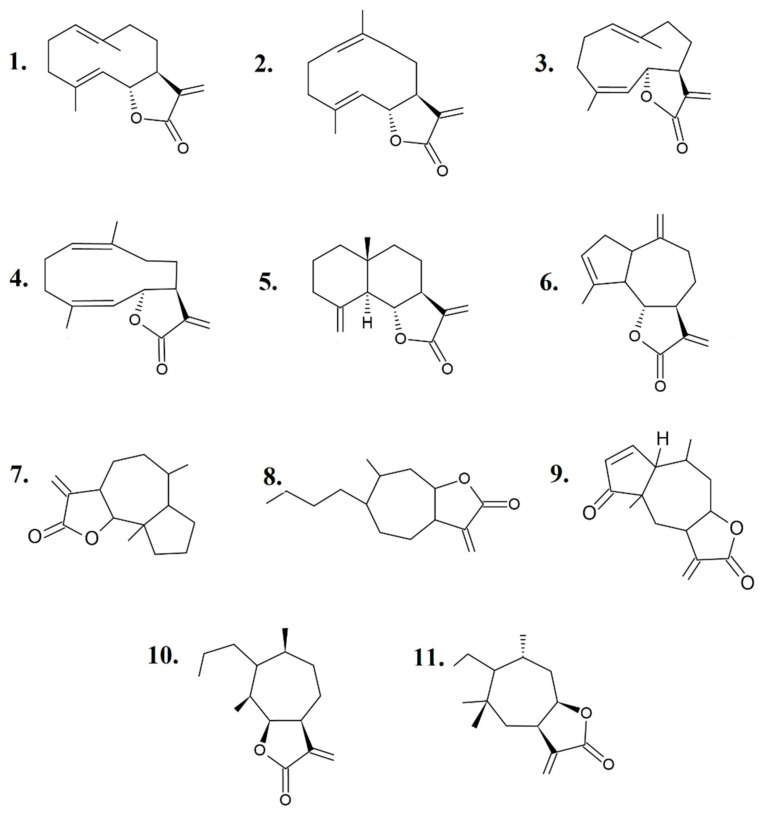
Skeletons of sesquiterpene lactones: Germacrolide (**1**), Melampolide (**2**), Heliangolide (**3**), *cis*,*cis*-Germacradienolide (**4**), Eudesmanolide (**5**), Guaianolide (**6**), Ambrosanolide (**7**), Xanthanolide (**8**), Helenanolide (**9**), Secoambrosanolide (**10**), and Secohelenanolide (**11**).

STLs are a type of terpenoid that contains 15 carbon atoms in an isoprenoid structure with a lactone function [2]. They are derived via the mevalonic acid pathway and consist of a typical five-membered or γ-lactone ring, containing an exocyclic methylene conjugated with the carbonyl group [3]. Owing to the presence of different functional groups, these chemical compounds are open for structural modifications and thus are counted as biologically significant entities. These alkylating agents can inhibit key enzymes and proteins in cells by forming covalent adducts and act as potent apoptotic inducers in several cancer cell lines [4]. The activities of STLs are noticeable at extremely low concentrations and depend on the lipophilicity, molecular geometry, and number of alkylating structures in the compound; the chemical environment; and the target sulfhydryl group [5,6]. STLs are classified into four major groups: germacranolides, eudesmanolides, guaianolides, and pseudoguaianolides; however, depending on the arrangement of their core skeletons, STLs may have many structural subtypes [1,2,7,8], (Figure 1).

In the past, several reviews have focused on the distribution, synthesis, physical, and biochemical properties of STLs [2,3,9,10,11]. Despite their cytotoxic nature, STLs have gained the attention of biologists and chemists and are accepted as lead molecules in the field of medicine. However, the biological activity of many STLs are still being evaluated, and assembling information on the individual compounds, which shows promising results, is essential. Parthenin is one such compound that has been tested for a wide range of pharmacological and pesticidal activities, and attempts have been made to diminish its cytotoxicity and to enhance its efficacy via structural modifications [12,13,14]. Therefore, an effort has been put forward to present an overview of parthenin through a comprehensive discussion. The objective of this review is to highlight various biological activities of parthenin, its multifaceted applications, and its associated limitations for future therapeutic and commercial applications.

## 2. Parthenin

**Figure 2:**
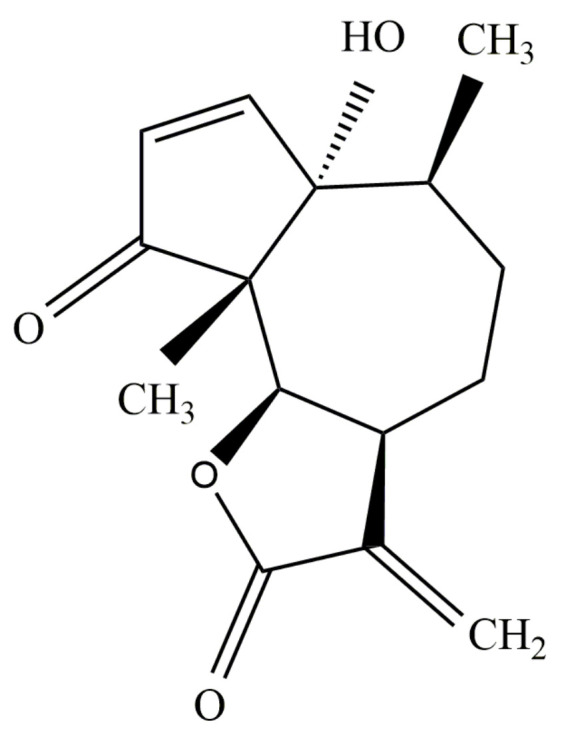
Structure of parthenin.

**Figure 3:**
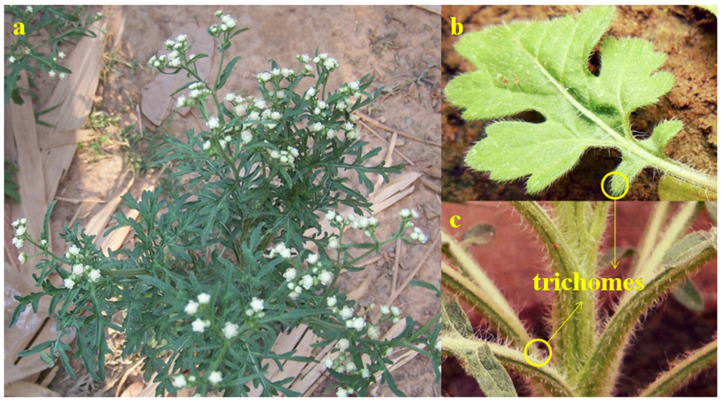
(**a**) *Parthenium hysterophorus* and the capitate-sessile trichomes present on its (**b**) leaves and (**c**) stem.

Parthenin (Figure 2), a pseudoguaianolide STL, is the major constituent of an invasive tropical weed *Parthenium hysterophorus* (ragweed parthenium; Asteraceae; Figure 3a) [15,16]. It is mainly sequestered in the capitate-sessile trichomes present on different parts of *P*. *hysterophorus* (Figure 3b,c), with the maximum amount being sequestered in the leaves [17]. Kanchan and Jayachandra [18] quantified the amount of parthenin present in the roots, stem, leaves, inflorescence, and fruits of the weed, which comes out to be 0.1%, 0.02%, 0.30%, 0.30%, and 0.15% on the dry weight basis, respectively. However, populations of *P*. *hysterophorus* in southern Bolivia, central Argentina, and Texas do not produce parthenin, but instead produce its diastereomer, hymenin [19]. The increased production of parthenin has recently been linked to elevated CO_2_ levels, with nearly 49% higher production at 400 ppm CO_2_ than at 350 ppm [16].

Parthenin is derived from the mevalonic acid pathway via the formation of farnesyl pyrophosphate, as is the case with other STLs. However, the exact pathway that differentiates the formation of parthenin from the other STLs is not yet fully understood. The biosynthesis of parthenin continues throughout the life of plant, with the maximum production observed during the reproductive stages [20]. Under natural conditions, it is either leached from the plant through ruptured trichomes and root exudates or released by decomposed tissues [21,22]. Due to the numerous biological activities of parthenin, its multi-step synthesis has also been observed [23,24].

Parthenin exhibits a multitude of activities, most of which are relatively less exploited. The use of *P*. *hysterophorus* in the traditional medicinal system of the Southeastern United States, West Indies, and Cuba for curing ulcerated sores, facial neuralgia, fever, and anaemia is due to the therapeutic properties imparted by parthenin [22,25,26,27]. At the same time, its presence in every part of the plant can be held accountable for public health issues such as contact dermatitis, asthma, allergenic responses, etc., as well as the bitter milk in cattle and livestock poisoning [28,29,30]. Apart from that, parthenin has been linked to the invasive success of *P. hysterophorus* by imparting unpalatability and allelopathy [31,32].

## 3. Structure of Parthenin

Parthenin (1,6-β-dihydroxy-4-oxo-10α*H*-ambrosa-2,11(13)-dien-12-oic acid-γ-lactone; 6α-hydroxy-6,9α-dimethyl-3-methylene-3,*3*α,4,5,6,6α,9α,9β-octahydro-azuleno(4,5-β)furan-2,9-dione) is a pseudoguaianolide STL with molecular formula: C_15_H_18_O_4_ and molecular weight: 262.305 g mol^−1^. Structurally, it is composed of a seven-membered ring assuming chair conformation and the two five-membered rings (cyclopentenone and lactone ring) assuming envelope conformations [33] (Figure 2). The presence of α-methylene-γ-lactone moiety and β-unsubstituted cyclopentenone ring along with five chiral centres is held responsible for its susceptibility to various biochemical groups and its wide spectrum of biological activities [31]. The presence of the two centres for the Michael addition of biological groups (α-methylene part of lactone moiety and the double bond of the cyclopentenone ring) impart alkylation properties to parthenin [34], which enables it to form adducts with –SH sulfhydryl compounds (e.g., cysteine and glutathione) [35,36]. This ability to react with –SH groups is non-specific and is of great biological significance as it increases the tendency of parthenin to react with the various nucleophiles, key enzymes, and factors involved in biological processes [37]. The presence of multiple reactive sites in the compound also provides a template for the structural modifications exploited by chemists and biologists for investigating further possibilities.

**Figure 4:**
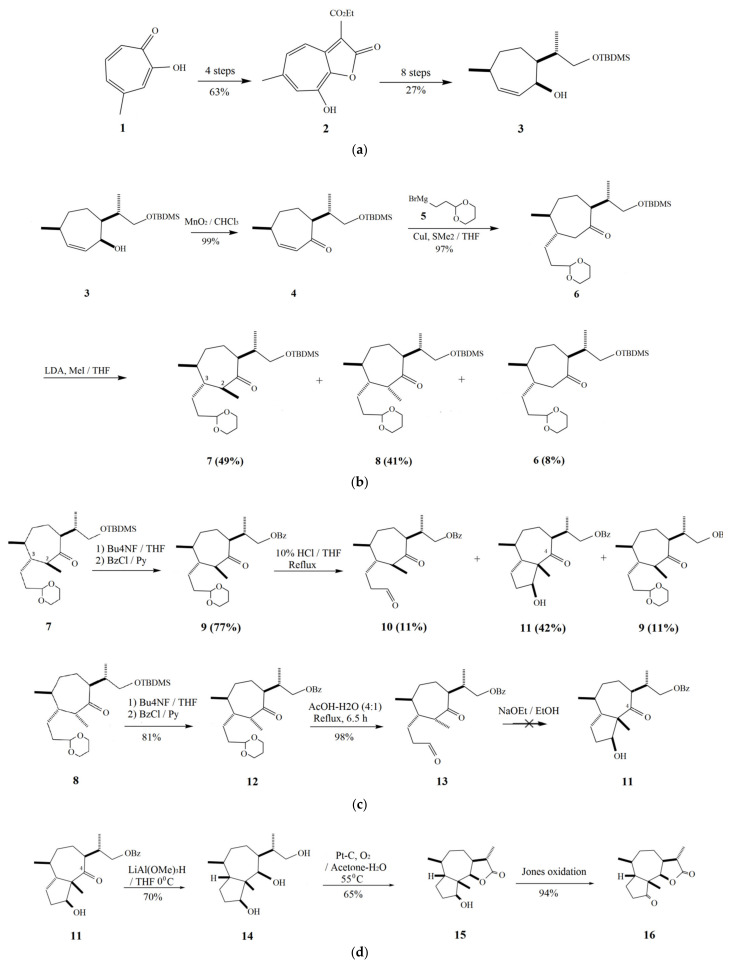

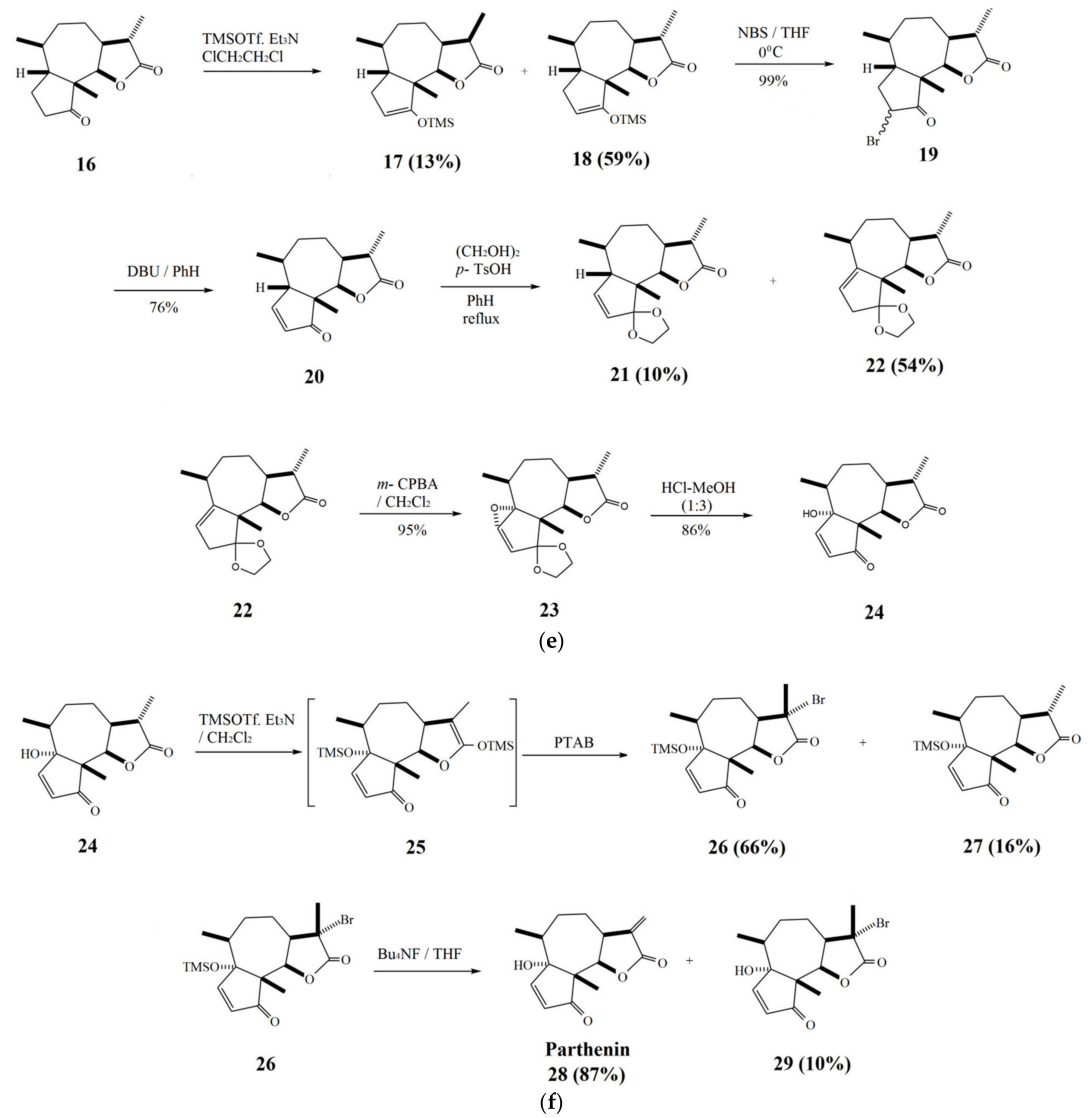
Synthesis of parthenin by the method provided by Shimoma et al. [24]. Compounds: 4-methyltropolone (**1**); ethyl 8-hydroxy-6-methyl-2-oxo-2*H*-cyclohepta[*b*]furan-3-carboxylate (**2**); cycloheptenol derivative (**3**); α,β-unsaturated ketone (**4**); Grignard reagent (**5**); α-oriented acetal (**6**); diastereomers (**7**,**8**); benzoates (**9**); intermediary aldehyde (**10**); aldol product (**11**); benzoates (**12**); aldehyde product (**13**); triol (**14**); γ-lactone alcohol (**15**); ketolactone (**16**); C-3 epimer (**17**); silyl enol ether (**18**); α-bromo ketone (**19**); α,β-unsaturated ketone (**20**); α,β-unsaturated ketal (**21**); ketal product (**22**); intermediate product (**23**); hymenolin (**24**); silyl enol ether (**25**); α-bromolactone (**26**); α-methyl-γ-lactone (**27**); parthenin (**28**); and 11 α-bromo hymenolin (**29**). (**a**) Formation of the key intermediate **3** from 4-methyltropolone (**1**) through another intermediate (**2**); (**b**) oxidation of **3**, followed by addition of Grignard reagent resulted in the formation of enone (**6**), methylation of which gave the intermediate **7** and its epimer **8**; (**c**) intramolecular aldol condensation of the intermediates **7** and **8** resulted in another intermediate **11**; (**d**) intermediate **11** is subjected to a reduction, forming triol **14**, which is then oxidised to yield **16** through **15**; (**e**) formation of the final intermediate, α-methylene-γ-lactone hymenolin (**24**) via lactonisation, oxidation, several protection/de-protection steps, dehydrogenation, epoxidation, and hydrolysis of **16**; and (**f**) hymenolin (**24**) is converted to parthenin (**28**) via α-bromination and dehydrobromination.

Parthenin can be extracted from its natural source, *P*. *hysterophorus*, using the powdered plant material; fractioned by preparative high-performance liquid chromatography with UV detection [38,39,40]; or produced synthetically via several methods, reviewed in detail by Barbero and Prandi [11]. The first total synthesis of racemic parthenin was performed by Kok et al. [23]. In this process, the key intermediate was obtained by photocycloaddition of 1,2-*bis*(trimethylsilyl-oxy)cyclopentene and 2-methyl-2-cyclopentanone. The intermediate was modified to produce neoambrosin, which was epoxidised to furnish parthenin along with hymenin. Heathcock et al. [29] introduced a multistep procedure involving the fusion of a five-membered ring onto a pre-existing cycloheptane precursor, which was then exploited for the enantioselective construction of a diastereomeric mixture, the epoxidation of which gave racemic parthenin. Asaoka et al. [41] performed enantioselective synthesis of parthenin using the trimethylsilyl group present on the unsaturated seven-membered ring. Another method of total synthesis of parthenin included using methyl tropolone, resulting in hymenolin as the final intermediate, which was converted to parthenin [24] (Figure 4).

## 4. Pharmacological Properties of Parthenin

Ethnobotanical studies have revealed the importance of *P. hysterophorus* in traditional medicinal systems in different parts of the world since antiquity for its antiparasitic, antibacterial, antifungal, amoebicidal, antimalarial, and febrifuge properties [26,27]. These pharmacological activities have also been verified using the extracts of *P. hysterophorus* [42,43,44,45,46]. Pharmacological research on parthenin emerged in the 1970s in Mexico when it was isolated from *P*. *hysterophorus* to evaluate its medicinal value [47]. Since then, pure parthenin, and its derivatives have been explored for their medicinal aspects.

### 4.1. Anti-Cancerous Activity

**Figure 5:**
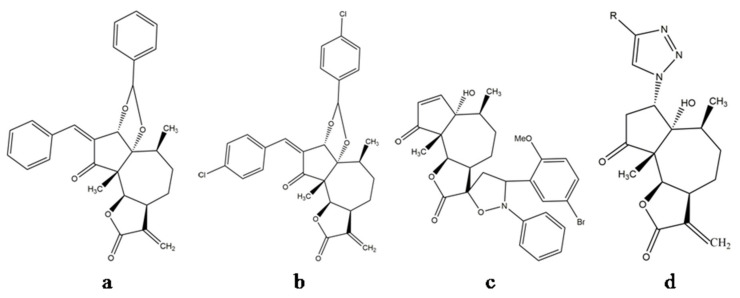
Compounds synthesised from parthenin: (**a**) P16, (**b**) P19, (**c**) SLPAR 13, and (**d**) 1,2,3-triazole derivatives of coronopilin.

In 1982, pure parthenin was described as a novel anti-cancerous lead by Mew and colleagues [48], who reported a significant reduction in tumour size and spread and an enhanced survival of the test species when the compound was assayed against tumour cell lines. This observation was strengthened by further investigations involving parthenin and its structural analogues. Parthenin and its derivatives have been found to exhibit cytostatic and anti-angiogenic potential [13], chemotherapeutic abilities [34], and anti-proliferative activities [43,49,50]. For example, analogue P16 (Figure 5a) inhibited human acute lymphoblastic leukaemia MOLT-4 cells [50] and pancreatic adenocarcinoma PANC-1, Mia PaCa-2, and AsPC-1 cells (IC_50_ = 3.4 μM) [13], and analogue P19 (Figure 5b) inhibited proliferation of human myeloid leukaemia (HL-60) cells (IC_50_ = 3.5 μM [43]). Several spiro-derivatives of parthenin (benzonitrile oxides, nitrones, and azides with an exocyclic double bond of C ring (α-methylene-γ-butyrolactone)) exhibited improved anti-cancerous activity against human cancer cell lines with low mammalian toxicity compared with parthenin [6]. SLPAR13 (Figure 5c), a spiro-isoxazolidine derivative of parthenin, caused cell death in three human cancer cell lines, namely HL-60, SiHa, and HeLa [51]. Khazir and co-workers found that 1,2,3-triazole derivatives of coronopilin (Figure 5d), synthesised from parthenin, were effective against PC-3 cell lines (IC_50_ value = 3.1 μM) as well as against the NF-κB (p65) transcription factor (with 80% inhibition in 24 h at 100 μM) [52]. These studies represent parthenin as a future chemotherapeutic drug; however, since most of these studies are limited to in vitro cultures or animal models, it is difficult to count upon these effects in humans [3].

### 4.2. Anti-Malarial Activity

Parthenin also exhibited significant anti-malarial activity against a multi-drug resistant strain of *Plasmodium falciparum* and a striking structural similarity with a new anti-malarial drug, qinghaosu, at the molecular level [53]. The activity of parthenin against *P*. *falciparum* was effective enough to potentially replace the artemisinin-related drugs in case of artemisinin-resistant parasites [54]. A docking analysis of parthenin analogues against lactate dehydrogenase proteins suggested that some ligands have excellent binding affinity against *P*. *vivax* and *P*. *falciparum*, and therefore, these may serve as drugs in anti-malarial therapy [55].

### 4.3. Others

Parthenin exhibited amoebicidal activity comparable with that of the standard drug metronidazole when tested against *Entamoeba histolytica* [56]. Parthenin was demonstrated to exhibit anti-inflammatory activity using the in vitro expression of TNF-α, IL-1β, and IL-6 in murine neutrophils [57]. Parthenin and its regio- and stereoselective derivatives exhibit antibacterial activity against different gram-positive and gram-negative organisms [58]. Despite these medicinal properties, the toxicity of parthenin is a major concern in its acceptance as a medicinal drug.

## 5. Phytotoxic Property of Parthenin

The allelopathic potential of *P*. *hysterophorus* has been well established, and the weed is known to have growth-retarding effects on a series of crops, weeds, and tree species [59,60,61,62]. Several studies revealed the key role of parthenin in imparting these allelopathic properties to *P*. *hysterophorus* [5,18].

The herbicidal properties of pure parthenin have been examined against *Ageratum conyzoides* [63], *Bidens pilosa*, *Avena fatua* [64], *Amaranthus viridis*, *Chenopodium murale* [65], *Cyperus rotundus* [66], and *Cassia tora* [12]. Both pre- and post-emergent application of parthenin affected seedling growth, dry weight, and photosynthesis in *Amaranthus viridis*, *Cassia occidentalis*, *Echinochloa crus-galli*, and *Phalaris minor* [67]. The concentrations of parthenin that affected the agricultural weed *A*. *conyzoides* did not seem to affect the crop *Triticum aestivum* [63], pointing towards the selective phytotoxicity of the compound. Different aquatic weeds were also reported to be affected by parthenin and it has also been proven lethal to certain submerged weeds (*Najas graminea, Ceratophyllum demersum,* and *Hydrilla verticillata*) at extremely low concentrations [68]. With more precise knowledge about the specific concentrations that selectively affect a particular weed, the duration of exposure, and the mode of treatment, parthenin can be successfully utilised for managing the uncontrolled growth of aquatic/agricultural weeds.

Further evaluation of such studies suggested a pattern of dose-dependent phytotoxicity in the test species [63,69]. Even though parthenin suppresses its competitors, certain interesting observations were made in *Phaseolus aureus*, *Sinapis arvensis*, etc., where growth stimulatory effects were seen at low concentrations upon the application of parthenin or its derivatives [21,70]. The activity of parthenin can be compared with that of Indole-3-acetic acid (IAA), a well-known growth regulator [70]. This indicates the possibility of developing suitable compounds/doses that could encourage “herbicide-related-hormesis” (an herbicide stimulating growth in crops along with weed management). Phytotoxins with an auxin=like mode of action or the anti-auxins that target auxin-mediated processes show such biphasic effects depending on the active concentrations and thus are being used as successful herbicides [71]. However, since this phenomenon largely depends on the growth conditions [72], more systematic studies are required to interpret its actual potential.

Most of the studies conducted to test the phytotoxicity of parthenin have proved it to be a potent root inhibitor [64,68,73]. The findings suggest that the compound may alter the contents of some macromolecules [5], modify the enzymatic activities of the plant cells [31], damage cell membrane [68], cause excessive electrolyte leakage [67], affect respiratory electron transport ability of embryo [69], or disrupt photosynthetic activity due to the loss of chlorophyll [64,68]. It may also react with the -SH group of amino acids and proteins via non-reversible alkylation and may change their characteristic behaviour, as generally seen in STLs [8]. Batish et al. [63] stated that the reduction in seedling length of *A*. *conyzoides* might have resulted due to the inhibition of the function of gibberellins and IAA.

However, the above-mentioned assumptions about its mode of action remain speculative and describe only the secondary or tertiary level of reactions by the plant system. Further detailing is required in this regard, particularly in reference to their action at molecular level. By knowing the exact mechanism of action, the phytotoxic effects of the compound can further be modified as per the requirements by using modern biotechnical/genetic engineering techniques. This could lead to the development of a much smarter, safer, and more functional series of herbicides.

## 6. Pesticidal Properties of Parthenin

### 6.1. Insecticidal Properties

Various insect species such as moths (*Phthorimaea operculella* and *Spodoptera litura* [74,75]), migratory grasshoppers (*Melanoplus sanguinipes* [76]), cotton stainers (*Dysdercus koenigii* [74]), mosquitos (*Aedes atropalpus* [77]), and stored grain pests (*Callosobruchus chinensis*, *Tribolium castaneum*, and *T*. *confusum* [74,78,79]) were affected upon exposure to the pure parthenin. Datta and Saxena [12] demonstrated the insecticidal and nematicidal activities of parthenin and its derivatives against the stored grain pest *Callosobruchus maculatus* and root knot nematode *Meloidogyne incognita*. Parthenin showed moderate repellent activity against the diamondback moth, *Plutella xylostella* (LC_50_ = 1709.42 mg L^−1^), whereas it was highly effective against aphid, *Aphis craccivora* (LC_50_ = 947.87 mg L^−1^; [80]). In a study testing the insecticidal potential of STLs, it was observed that, along with parthenin, helenalin and coronopilin were found to reduce the survival of the confused flour beetle, *Tribolium confusum*, at concentrations higher than 3%, whereas another pseudoguaianolide, Tenulin, had no significant effect. This could be attributed to the absence of α-methylene-γ-lactone moiety, which was otherwise present in the remaining three lactones [78]. Studies trying to explore the possible action mechanisms of parthenin in insects also concluded that the lethal effects could possibly be due to the cardiac inhibiting properties generated by the interference of α-methylene-γ-lactone moiety present in parthenin with free –SH groups [76,78].

### 6.2. Fungicidal Properties

Parthenin was found to inhibit the sporangial germination and zoospore motility in various plant pathogens such as *Sclerospora graminicola*, *Pestalotia* sp., *Cladosporium herbarum*, *Helminthosporium sativum*, *Curvularia lunata*, etc., indicating its fungicidal tendencies [81,82]. As a fungicidal compound, it has been reported to cause lobulations, hyphal wall thickening, and restricted mycelial growth, and the effect was comparable to polyene antibiotics [82].

## 7. Toxicological Concerns

The toxicity–bioactivity relationships in parthenin are quite a subject of interest. The toxicity of parthenin is a major hindrance to its medicinal use. The unpalatability of its constituent weed, *P*. *hysterophorus,* is attributed to the presence of parthenin [32]. Even *Zygogramma bicolorata*, a biocontrol agent of *P*. *hysterophorus,* avoids plants/plant parts that are rich in parthenin [83]. The compound is compartmentalised into the glandular trichomes of *P*. *hysterophorus* to prevent autotoxicity in the plant [2].

Parthenin is the major antigen responsible for the incidence of contact dermatitis in humans upon exposure to *P*. *hysterophorus* [19,28,84]. The different patterns include classical airborne contact dermatitis, chronic actinic dermatitis, and mixed-pattern dermatitis [85]. Allergenic reactions are generally thought to be induced by the exocyclic α-methylene-γ-lactone moiety, but the mechanisms could vary depending on the antibody’s specificity to the non-functional groups present in the compound [3]. Of late, it has been concluded that parthenin induces oxidative stress and inflammatory responses in humans by changing the mRNA expression of proinflammatory cytokines, IL-1β, and IFN-γ via the activation of NF-κB [86].

The clastogenic effects of parthenin have been observed in terms of chromosomal aberrations (mainly chromatid breaks) and nuclear alterations such as pycnosis, micronuclei, and karyorrhexis in the animal tissues, which are the outcomes of interrupted cytokinesis, nuclear restitution, and DNA replication [26]. Nearly 50% inhibition in RNA, DNA, and protein synthesis and deterioration in the activities of key cellular enzymes were observed after 24 h of treatment with 1 µg mL^−1^ of parthenin [87]. Its interference with mitochondrial oxidative phosphorylation is also evident in certain cases [88]. A mixture of parthenin with another STL, coronopilin, modulated the afferent neurons and murine tracheal rings [89]. A strong correlation was observed between parthenin, and cytotoxicity induced by *P. hysterophorus* extracts in mouse fibroblast cell suspension [90]. The level of cytotoxicity was enhanced when the cells were exposed to UV-A radiation, which suggests that the compound may cause photosensitisation in animals and humans [90].

The toxic effects of parthenin have also been observed in plants. It leads to the degradation of chlorophyll, protein, and carbohydrate contents (and their de novo synthesis); the inhibition of respiration; and alteration in the activities of proteolytic and carbohydrate metabolising enzymes [5,67,69] Parthenin also caused light-dependent electrolyte leakage in the leaves of certain plant species, which suggests a disruption in the membrane permeability [67]. The mutagenic and cytotoxic effects of parthenin have also been observed in the plant tissues [91].

Therefore, before successfully introducing this compound as a therapeutic drug or an alternative to the synthetic pesticides, its toxic effects need to be studied with more specificity by undertaking long-term studies to ensure the safety of these products towards human/livestock health.

## 8. Prospects and Way Forward

There is a strong possibility of exploiting parthenin in different scientific fields, e.g., chemistry, medicine, and agriculture; however, toxicity of the compound is the major hindrance to its applications. To overcome these constraints and to improve the efficacy of its bioactivities, the following suggestions can be employed:

### 8.1. Development of Structural Analogues

**Figure 6:**
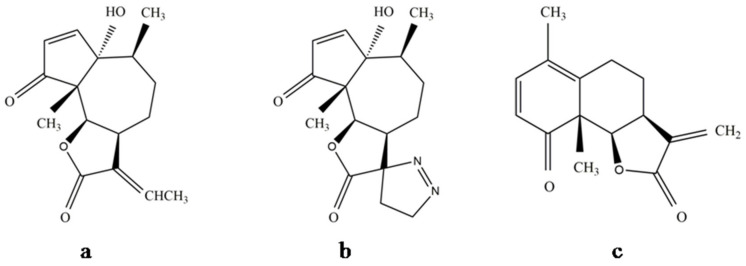
(**a**) Propenyl derivative of parthenin, (**b**) pyrazoline adduct of parthenin, and (**c**) rearranged product of parthenin.

**Figure 7:**
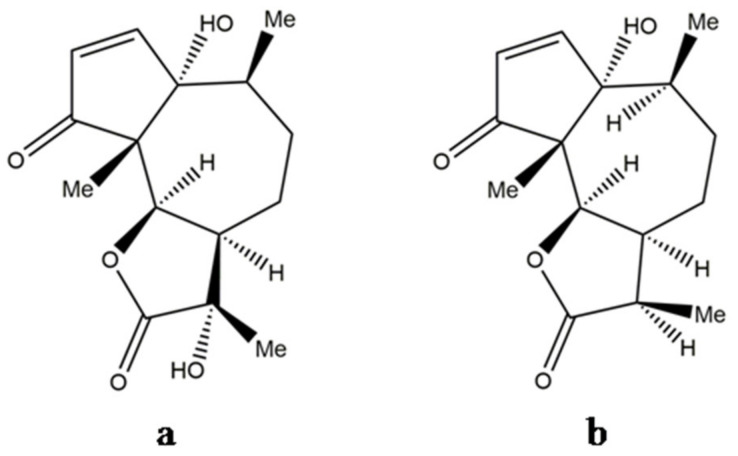
(**a**) C-11 hydroxylation product of parthenin and (**b**) C-11 reduction product of parthenin.

Obtaining suitable derivatives through structural modifications may reduce the levels of toxicity and may enhance the effectiveness of the compound. Biochemical changes in the structural properties of parthenin were found to alter its growth regulatory actions [70]. As already discussed in Section 4, Section 5, and Section 6, several parthenin derivatives have been tested along with the compound for pharmacological and pesticidal applications. Some studies have shown that the performance and safety profile of these derivatives are relatively much better when compared with the key metabolite of *P. hysterophorus*. It has been suggested that monofunctional alkylants generally have less side effects, and therefore, generating the monofunctional analogues of parthenin could overcome its toxicity-based limitations [6]. The 1,3-dipolar cycloaddition of diazomethane to parthenin gave complete chemoselectivity and 81% of diastereoselectivity in favour of the (11*S*)-stereoisomer to its spiropyrazoline [92]. Datta and Saxena [12] observed that the saturated lactone derivative of parthenin was 2.25 times more active than parthenin, and the other modified compounds, e.g., propenyl derivative (Figure 6a), pyrazoline adduct (Figure 6b), and a rearranged product of parthenin (Figure 6c), were proven to be more effective herbicides, insecticides, and nematicides, respectively. A C_16_-derivative of parthenin is reported to enhance the growth and development in maize and mung bean [93]. Recently, two derivatives, namely, ethylene glycol derivative and 2a-azidocoronopolin, synthesised by derivatisation of the α,β-unsaturated carbonyl group of parthenin through the addition of hydroxyl groups, were found to exhibit 2−4-fold higher larvicidal effects against the African malaria vector, *Anopheles gambiae* [14]. Similarly, certain derivatives of parthenin exhibited improved pharmacological properties compared with parthenin [6,34]. Endocyclic unsaturation of parthenin through regioselective Baylis Hillmann adducts resulted in analogues with significantly reduced cytotoxicity, which implies that tampering with the pharmacophoric cyclopentenone ring structure results in the loss of the NF-κB binding by the ligand and inhibition of telomerase [34]. Microbiological transformation is another method of introducing substituents into the carbon skeleton of parthenin without disturbing the chromophores, and such efforts were made with the fungi *Sporotrichum pulverulentum* and *Beauveria bassiana*. This yielded a C-11 hydroxylation product (Figure 7a) and a C-11 reduction product (Figure 7b) of parthenin, respectively, with entirely different properties [94]. Such modifications can provide an effective solution to the limitations encountered, particularly with respect to toxicity and efficiency.

### 8.2. Selection of Suitable Doses

As it is said that dose is mainly responsible for deciding the toxicity of a substance, the most important aspect to be determined before exploiting parthenin as a medicinal drug or pesticide is the concentrations that are apparently safe. Toxic substances can be stimulatory or beneficial at low doses, as seen in most pharmaceutical drugs [95]. An assortment of the non-toxic concentrations of parthenin not only enhances its application as a pharmaceutical product or pesticide but also may promote “herbicide-related-hormesis”, as discussed earlier in Section 5.

### 8.3. Characterisation of Parthenin

Before deciding the applications of a compound, it is imperative to understand how it behaves under different environmental conditions. Parthenin has been researched for its phytochemical properties, but only a few studies have addressed the issue of the accumulation and stability of parthenin in the environment. The question is important from two different perspectives, as it will decide its efficacy as a pesticide and the extent of its environmental impact. According to Pandey [68], the toxicity of parthenin persisted for 30 days in an aquatic environment. In another study, Belz and co-workers tried to investigate the fate of parthenin in soil and found it to be governed by several physiochemical and biological processes [32]. Soil sterilisation and low soil moisture slowed down the degradation of the compound, whereas high temperature, soil preconditioning with parthenin, the clayey content of soil, and parthenium infestation accelerated the process. Rajiv and co-workers also confirmed the eradication of parthenin by earthworms and microbes through vermicomposting [96]. However, these studies were undertaken in variable ecological conditions and, hence, cannot be compared to draw a conclusive verdict. Thus, quantitative results with more certainty are demanded in this regard.

### 8.4. Others

Protocols regarding the extraction process of parthenin also need to be simplified to ensure its wider applicability. Batish et al. [63] suggested that the production of the compound should be enhanced via tissue culture or other biotechnological approaches. Apart from this, not much is known about its interaction with various biotic and abiotic components present in the environment.

## 9. Conclusions

In conclusion, it can be said that parthenin is a novel, unexploited, and undermined molecule that, despite being characterised by a multitude of properties, is neglected due to the lack of systematic studies. A large part of this negligence is attributed to the toxic properties of parthenin, which is a primary factor limiting its applicability. This is especially true in the case of pharmacological aspects, which mainly depend on the toxicity–bioactivity relationship of a compound. However, there is much evidence in the literature that states the possibility of overcoming the toxic nature of parthenin by thoroughly understanding its structural basis, designing suitable derivatives, and deciding the appropriate doses. At the same time, it is also important to look for unanswered questions pertaining to its synthesis, stability, and impact on environment. By focusing on these aspects, meticulously understanding the loopholes, expanding the research in wider directions, and utilising recent biotechnical advancements, it is feasible to develop a range of apposite derivatives, drugs, pesticides, and biological substitutes that could be exploited for multipurpose activities from this versatile chemical compound, parthenin.

## Data Availability

Not applicable.
